# Seasonal and 2009 Pandemic H1N1 Vaccine Acceptance as a Predictor for COVID-19 Vaccine Acceptance

**DOI:** 10.7759/cureus.21746

**Published:** 2022-01-30

**Authors:** Priya Nair, Danielle P Wales

**Affiliations:** 1 Medicine, Albany Medical College, Albany, USA; 2 Health Policy, Management and Behavior, University at Albany School of Public Health, Rensselaer, USA; 3 Medicine, Division of Internal Medicine/Pediatrics, Albany Medical Center, Albany, USA

**Keywords:** acceptance, influenza, hesitancy, vaccine, covid-19

## Abstract

The emergence of COVID-19 also began an unprecedented production and distribution of several novel COVID-19 vaccines to combat the pandemic. Unfortunately, with the history of vaccine hesitancy in the United States and abroad, concern remains regarding the ability to vaccinate enough of the population to achieve herd immunity. In this study, 101 adults were surveyed about their vaccine experience in the waiting room of their visit to a Med-Peds clinic in Albany County, NY, to gauge interest in the upcoming rollout of COVID-19 vaccines. Questions included their opinions on seasonal influenza vaccines, the 2009 H1N1 vaccine, and the COVID-19 vaccine. The results of our survey are consistent with previous studies where gaps in acceptance were notable in black populations, lower education, and individuals with public health insurance. Furthermore, 92.9% of respondents who denied getting the 2009 H1N1 pandemic vaccine also did not plan to receive the COVID-19 vaccine (p<0.05), therefore a strong correlation was found between receipt of the previous 2009 H1N1 pandemic vaccine and the COVID-19 vaccine acceptance. The qualitative results of our study revealed that COVID-19 precautions deterred people from receiving the seasonal influenza vaccine, and a lack of information on the COVID-19 vaccine caused hesitancy to receive the vaccine on behalf of patients.

## Introduction

With 50 million cases in the United States and 812,000 deaths as of December 7, 2021, the need for COVID-19 vaccination is evident. There are currently three available COVID-19 vaccines in the United States [1]. Despite the availability of these three vaccines, there is cause for concern regarding how much of the American population will accept COVID-19 vaccination. Influenza is a respiratory viral illness with significant morbidity and mortality annually [2]. Although influenza vaccination is recommended by the CDC for all persons six months of age and older, uptake has been historically poor. In 2019, only 34% of people aged 18-49 received the influenza vaccine [2]. One study that assessed the perceptions of seasonal vaccination among healthcare workers identified some topics of hesitancy. This included the perception of influenza as a mild disease, believing that their immune systems were “strong,” and they did not identify themselves as a risk group needing added protection against the disease [3]. Similarly, many people feel the COVID-19 virus causes mild disease. The misinformation and lack of knowledge continue to be a barrier as the COVID-19 vaccine begins to come into question. Other hesitancies that may arise include refusal to get either vaccine due to the perceived protection from mandatory mask policies [4].

Multiple previous studies have shown a strong correlation between seasonal influenza vaccine rates and the pandemic H1N1 vaccine in 2009 [5,6]. In fact, the CDC reported a huge spike in seasonal influenza vaccinations after the 2009 pandemic [7]. Despite the increase in seasonal influenza vaccinations, in the wake of the 2009 pandemic, many people still had reservations about receiving their influenza vaccines. During the 2009 pandemic, 64.4% of interviewed college students believed that there are negative side effects to the H1N1 vaccine [8]. In 2011, demographic and economic reasons were found to be strong indicators of influenza vaccine adherence [9]. One study found a more pronounced relationship between non-adherence and lower economic status [10].

Previous studies on the influenza vaccine have not included the effect of masking and social distancing policies, which have been an important public health intervention during the COVID-19 pandemic. Therefore, it is possible that there are alternative motivators specific to the influenza vaccine during the 2020-2021 influenza season that should be noted. Reviewing factors that promote or hinder immunization from the patient's perspective during this current pandemic may decrease decisional conflict and increase vaccination rates.

We hypothesize that the survey will identify correlations between the uptake of seasonal influenza, 2009 H1N1, and COVID-19 vaccines as well as disparities in terms of influenza vaccine adherence that could potentially be addressed as the COVID-19 vaccine rollout continues.

## Materials and methods

Study design

This protocol was reviewed and approved by the Albany Medical Center Institutional Review Board (6083). From February 8 to February 23, 2021, a total of 101 adult patients aged 18 and above were approached in the waiting room of the Albany Medical Center Internal Medicine/Pediatrics practice with the opportunity to participate in an anonymous survey, accessible by paper or electronically via Qualtrics®. Participation was voluntary, and participants did not receive any incentives for their participation. A copy of the survey is shown in Appendix 1. Survey questions were directly adapted from previously validated studies [11-17]. No personally identifiable information was obtained from the surveys.

Inclusion and exclusion criteria

All patients over the age of 18 in the waiting room of the Albany Med-Peds office were approached and offered the survey. Because this population has full autonomy over their medical care, it will be beneficial for gaining insight into their decisions. Participation in the survey was voluntary. The survey was offered in English, so any patient with language barriers was excluded.

Data analysis

Survey responses were numbered and the data was entered into an Excel spreadsheet by the student investigator. No identifying information was present. Results were analyzed using Stats iQ™ via Qualtrics®. Comparison of proportions was performed using Chi-square.

## Results

Demographic data were gathered on race, ethnicity, gender, religion, education level, health insurance, occupation, and zip code (Table 1). In addition, the survey asked about attitudes and behaviors toward annual influenza, 2009 H1N1, and COVID-19 vaccines. Any data regarding ethnicity, religion, occupation and zip code yielded no significant data and was omitted from the results. Any specific data that was found to be significant (p<0.05) contains an asterisk.

**Table 1:** Demographic data.

Mean (age)	n=100
18-19	9
20-24	20
25-29	9
30-34	13
35-39	6
40-44	6
45-49	8
50-54	8
55-59	4
60-64	4
65-69	4
70-74	3
75-79	3
80-84	1
85-89	2
Education	n=101
Less than high school	8
High school graduate	17
Some college	22
2 year degree	4
4 year degree	27
Graduate degree	21
Doctorate degree	2
Race	n=101
American Indian or Alaskan	1
Asian	11
Black or African American	18
White	67
Other	3
Ethnicity	n=97
Hispanic/Latino	7
Non-Hispanic/Latino	75
Prefer not to answer	15
Gender	n=100
Male	46
Female	53
Non-binary/Third gender	1
Education	n=101
Less than high school	8
High school graduate	17
Some college	22
2 year degree	4
4 year degree	27
Graduate degree	21
Doctorate degree	2
Insurance	n=101
Private	57
Public (Medicaid, managed Medicaid, Medicare, managed Medicare)	42
Prefer not to answer	2

The data indicate a significant correlation between consistent influenza behavior and agreeability with the COVID-19 vaccine (Table 2). Similarly, there is a significant correlation between poor influenza vaccine uptake and disagreeability with the COVID-19 vaccine. Of those that consistently receive the influenza vaccine before every season, 93.5% of respondents agreed to get the COVID-19 vaccine. Whereas, 68.8% of respondents who have never received the influenza vaccine disagreed with receiving the COVID-19 vaccine. In addition, a significant correlation between hesitancy toward the previous H1N1 vaccine and the current COVID-19 vaccines was observed (Table 3). Of those who did not receive the 2009 H1N1 vaccine, 92.9% of them disagreed with getting the COVID-19 vaccine. Similarly, there was a significantly low number of respondents (26.5%) that agreed to get the COVID-19 vaccine despite denying the 2009 H1N1 vaccine. On the other hand, of those that chose to receive the 2009 H1N1 vaccine, 26.5% of them intended to receive the COVID-19 vaccine.

**Table 2:** COVID-19 vaccine acceptance compared to seasonal flu vaccine acceptance.

	If the Covid-19 vaccine becomes available, I will get it.				
What best describes your flu vaccination behavior? N = 101	Agree	Disagree	Unsure	Chi-Square	P-value
I get the flu shot before every season	93.5%*	N/A	6.5%*	66.4	<0.00001
I’ve gotten a flu shot once in a while	51.6%	12.9%*	35.5%		
I get a flu shot if they predict a serious flu outbreak	50.0%	N/A	50.0%		
I’ve never gotten a flu shot	5.7%*	68.6% *	25.7%		

**Table 3:** COVID-19 vaccine interest compared to 2009 pandemic influenza vaccine self-reported receipt.

Did you receive the 2009 H1N1 Vaccine? n=100	If the Covid-19 vaccine becomes available, I will get it.				
	Agree	Disagree	Unsure	Chi-Square	P-value
Yes	26.5%*	7.1%	4.2%	34.9	<0.00001
No	26.5%	92.9%*	62.5%		
I do not remember	46.9%*	N/A	33.3%		

There is a significant correlation between the level of education and agreeability to receive the COVID-19 vaccine (Table 4). Those with education levels less than high school (12.5%), high school (23.5%), and some college (27.3%) had a significantly less percentage of respondents indicating they would get the COVID-19 vaccines than those with higher levels of education such as a four-year degree (70.4%) and a graduate degree (71.4%). Similarly, there is a significant correlation between those with private healthcare insurance and agreeability to receive the COVID-19 vaccine. Respondents with private health insurance responded at a much higher percentage (59.6%) to agree to receive the COVID-19 vaccine than those with public health insurance (33.3%).

**Table 4:** Demographic data compared to COVID-19 vaccine acceptance.

Education n=101	If the Covid-19 vaccine becomes available, I will get it				
	Yes (%)	No (%)	Unsure	Chi-Square	P-value
Less than high school	12.5%*	62.5%*	25.0%	32.8	0.00103
High School Graduate	23.5%*	41.2%	35.3%		
Some college	27.3%*	45.5%*	27.3%		
2 year degree	50.0%	N/A	50.0%		
4 year degree	70.4%*	3.7%*	25.9%		
Graduate degree	71.4%*	23.8%	4.8%*		
Doctorate degree	100%	N/A	N/A		
Insurance n=101	Yes	No	Unsure	9.01	0.0609
Private	59.6%*	17.5%*	22.8%		
Public (Medicaid, Managed Medicaid, Medicare)	33.3%*	40.5%*	26.2%		

Table 5 describes why people were hesitant and chose not to receive the influenza vaccine this year. The breakdown indicates that most respondents do not trust its efficacy. The next popular responses included reasons related to precautionary COVID-19 measures as protection, apathy, and skepticism toward the risk of influenza. Table 6 describes the optional qualitative response regarding comments on the COVID-19 vaccine. The primary reason people are hesitant is that they would like more information. Several respondents did not believe the disease caused by the virus was severe or trusted that their natural immunity was sufficient. Although the respondents were not prompted to give a positive or negative response, almost all written responses were negative.

**Table 5:** Reasons given by patients for not taking 2020-2021 influenza vaccine.

Reasons why people chose not to get the flu vaccine this year	
Reasons	Sample size (n=34)
Do not trust its efficacy	26%
Do not feel they need it	21%
Didn’t feel the need with COVID precautions	21%
Unclear ingredient/ Risks	14.2%
No time or money	8.8%
Not an issue	8.8%

**Table 6:** Qualitative comments on COVID-19 vaccine.

Reasons	Sample Size (n=42)
Needs more information	26%
Feels protected from having COVID	19%
COVID is not a major issue	19%
Unmotivated due to poor vaccine rollout	14%
Already received the vaccine	7.1%
Feels it is unsafe	7.1%
Other	7.1%

## Discussion

The main results of our study included strong correlations between seasonal influenza vaccine and COVID-19 vaccine adherence, and 2009 H1N1 vaccine and COVID-19 vaccine adherence. In our study, if respondents agreed to get the influenza shot before every season, they were more likely to get the COVID-19 vaccine. Similarly, if they have never gotten the influenza shot, they were less likely to get the COVID-19 vaccine (Table 2). Pastorino et al. showed similar results when assessing university students and their willingness to get both seasonal influenza and COVID-19 vaccine and found them to be strongly and positively correlated [18]. Additionally, the respondents of our study that received the 2009 H1N1 vaccine were more likely than not to receive or plan to receive the COVID-19 vaccine. Similarly, out of the people who did not receive the 2009 H1N1 vaccine, they were more likely to not express interest in the COVID-19 vaccine. This indicates a strong correlation between the 2009 H1N1 vaccine adherence and the current COVID-19 vaccine intent (Table 3). This trend has not been previously discussed in the literature.

In our study, individuals with less than a college degree, as well as those with public health insurance reported significantly less willingness to receive a COVID-19 vaccine (Table 4). Khubchandani et al. showed similar results when assessing COVID-19 vaccine acceptance and attributed these results to historical trends in vaccine hesitancy in these demographic groups. They also showed similar results when assessing COVID-19 vaccine acceptance within these demographics, noting that these demographics have a historically lower adherence toward vaccinations [19].

Specifically for the 2020-2021 influenza season, the main reasons individuals in our study did not receive the influenza vaccine included issues concerning efficacy and because of the new COVID-19 precautions (Table 5). Khubchandani et al. revealed similar results when assessing factors COVID-19 vaccine acceptance indicating that many people were less willing to get vaccines due to precautions they took during the 2020-2021 influenza season [19]. Overall, the main commentary people had regarding the COVID-19 vaccine was that they needed more information on it (Table 6). Although not prompted to give a positive or negative opinion regarding the vaccine, most of the respondents who chose to respond had a negative opinion. Some of the responses in the other category included questions regarding the comparative efficacy of the different vaccines. Despite most of the responses in this section expressing uncertainty around the vaccine, some responses expressed praise toward the vaccine.

There are some notable limitations. Our study had a relatively small sample size that represents the population served by an academic Med/Peds practice in Northeastern New York. Because the survey was entirely optional, there were not an equal number of responses across all questions. It is possible that many respondents experienced recall bias when answering about the 2009 H1N1 vaccine. Although there was an option to indicate if a person had trouble recalling their experience, this could have influenced the results of our study. However, since it was a novel vaccine at the time, most recipients would likely remember receiving it. In addition, at the time of our study, the COVID-19 vaccine was in the process of rollout, so some responses included issues with the availability of the COVID-19 vaccine, but that is no longer an issue at the time of publication. However, it is possible the negative attitudes toward rollout could have manifested into lasting hesitation toward the vaccine in general which can be studied in future studies. In addition, further studies could attempt to get the public opinion of how people felt the effectiveness of the vaccine was after getting it. Our study found consistent disparities in education and hesitancy in certain ethnic groups and populations. Future studies can work toward understanding and addressing these concerns among these more hesitant populations.

## Conclusions

In conclusion, our study was able to find a strong correlation between the 2009 H1N1 vaccine and COVID-19 vaccine hesitancy. In addition, we confirmed correlations between seasonal influenza vaccine and 2009 H1N1 vaccine hesitancy, as well as seasonal influenza vaccine and COVID-19 vaccine hesitancy. Our study also confirmed significant vaccine hesitancies in black, low education, and public health insurance populations regarding their willingness to get the COVID-19 vaccine. This indicates that behavior regarding the seasonal influenza vaccine can act as a predictor for the COVID-19 vaccine. Efforts to target these populations for education regarding the COVID-19 vaccine will be instrumental in improving vaccination rates and reducing the burden of COVID-19 on these populations.

## Appendices

Appendix 1. Survey Instrument

Albany Medical College Survey

1.     What is the purpose of this study?

The goal of this project is to understand any potential barriers to seasonal vaccination. A secondary object is to be conscious of any areas of hesitancy or uncertainty that comes with vaccination. We are hoping to understand the current perceptions around seasonal vaccines and vaccinations in general in order to better advocate for patients.

2.     Who is doing the study?

The team at Albany Medical College.

3.     What will happen if I participate?

At any point during the survey, you may decide that you no longer wish to continue. You do not have to continue the participation if you chose not to. You do not have to give a reason why you do not want to take part in the project. You will still be able to seek services at the Albany Medical College Department of Internal Medicine and Pediatrics. Your participation is voluntary.

D. How much time will this survey take?

We estimate that it will take about 5 minutes (2 pages back and front) to complete this survey.

If you have any questions about the study, you can email Priya Nair at .

Age (Years)  __________________

Race

❏   American Indian or Alaskan Native

❏   Asian

❏   Black or African American

❏   Native Hawaiian or Pacific Islander

❏   White

❏   Other_________________

❏   Prefer not to answer

Ethnicity

❏   Hispanic or Latino

❏   Not Hispanic or Latino

❏    Prefer not to answer

Gender

❏  Female

❏  Male

❏  Prefer Not to Answer

❏   Other____________

Religion

❏   Christian

❏   Judaism

❏   Islam

❏   Buddhism

❏   Hinduism

❏   Prefer not to answer

❏   Other____________________

Highest Level of Education Completed

❏   Less than high school

❏   High School

❏   Some College

❏   College Degree

❏   Graduate Degree

❏   Doctorate Degree

What type of health insurance do you have?

❏        Private

❏        Public (Medicaid, Managed Medicaid, Medicare, Managed Medicare)

❏        No Insurance

❏        Prefer not to answer

Current Occupation _________________________________

Current Zip Code _______________________________________________

Do you feel sufficiently informed about the benefits of the flu vaccine?

❏        YES

❏        NO

Do you feel sufficiently informed about the risks of the flu vaccine?

❏        YES

❏        NO

Did you get the flu vaccine this year?

❏        YES

❏        NO, BUT INTEND TO

❏        NO

What best describes your flu vaccination behavior?

❏        I get the flu shot before every season

❏        I’ve gotten a flu shot once in a while

❏        I get a flu shot if they predict a serious flu outbreak

❏        I’ve never gotten a flu shot

If you have received the flu vaccine, whose advice encouraged you to receive it?

_________________________________

If you are choosing not to receive the flu vaccine this year, can you provide the reason(s) for your decision?

________________________________________

Did you receive the 2009 H1N1 vaccine?

❏      Yes

❏      No

❏      I do not remember

Indicate if you agree with this statement:

If a COVID-19 vaccine becomes available, I will get it

❏        Agree

❏        Disagree

❏        Unsure

Any Additional Comments on the COVID-19 Vaccine ________________________________________________________________________________________________________________________________________________

Thank you for taking the time to complete this survey. Results will be kept confidential and will be used to learn more about the health of our population. The only right answer is your honest opinion!
